# Plasma membrane SK2 channel activity regulates migration and chemosensitivity of high‐grade serous ovarian cancer cells

**DOI:** 10.1002/1878-0261.13631

**Published:** 2024-03-13

**Authors:** Olivier Romito, Aude Lemettre, Aurélie Chantôme, Ophélie Champion, Noémie Couty, Lobna Ouldamer, Nadine Hempel, Mohamed Trebak, Caroline Goupille, Marie Potier‐Cartereau

**Affiliations:** ^1^ Inserm UMR 1069, Nutrition Croissance Cancer, Faculté de Médecine Université de Tours Tours France; ^2^ Réseau Molécules Marines, Métabolisme et Cancer and Réseau CasTHOR Cancéropôle Grand Ouest Tours France; ^3^ CHRU de Tours, Service de Gynécologie et d'Obstétrique Tours France; ^4^ UPMC Hillman Cancer Center, Division of Hematology & Oncology, Department of Medicine University of Pittsburgh PA USA; ^5^ Department of Pharmacology and Chemical Biology, Vascular Medicine Institute University of Pittsburgh PA USA

**Keywords:** cell migration, chemosensitivity, high‐grade serous ovarian cancer, LPA, SK2 channel, Taxol®

## Abstract

No data are currently available on the functional role of small conductance Ca^2+^‐activated K^+^ channels (SKCa) in ovarian cancer. Here, we characterized the role of SK2 (KCa2.2) in ovarian cancer cell migration and chemosensitivity. Using the selective non‐cell‐permeant SK2 inhibitor Lei‐Dab7, we identified functional SK2 channels at the plasma membrane, regulating store‐operated Ca^2+^ entry (SOCE) in both cell lines tested (COV504 and OVCAR3). Silencing *KCNN2* with short interfering RNA (siRNA), or blocking SK2 activity with Lei‐Dab7, decreased cell migration. The more robust effect of *KCNN2* knockdown compared to Lei‐Dab7 treatment suggested the involvement of functional intracellular SK2 channels in both cell lines. In cells treated with lysophosphatidic acid (LPA), an ovarian cancer biomarker of progression, SK2 channels are a key player of LPA pro‐migratory activity but their role in SOCE is abolished. Concerning chemotherapy, SK2 inhibition increased chemoresistance to Taxol® and low *KCNN2* mRNA expression was associated with the worst prognosis for progression‐free survival in patients with serous ovarian cancer. The dual roles of SK2 mean that SK2 activators could be used as an adjuvant chemotherapy to potentiate treatment efficacy and SK2 inhibitors could be administrated as monotherapy to limit cancer cell dissemination.

## Graphical abstract

Functional plasma membrane and intracellular SK2 channels participate in cell migration of serous ovarian cancer cells. Plasma membrane SK2 is a key player of cell migration induced by lysophosphatidic acid (LPA), an ovarian cancer biomarker of progression. Under LPA, the SK2 current increases, but the channel no longer contributes to the SOCE. Furthermore, the activity of plasma membrane SK2 channels promotes Taxol® sensitivity.

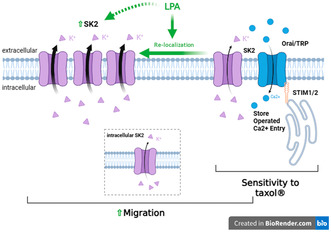

**Abbreviations**
K^+^
: potassium
Ca^2+^
: calcium
SKCa: Ca^2+^‐activated K^+^ channels
SOCE: store‐operated Ca^2+^ entry
CCE: constitutive Ca^2+^ entry
LPA: lysophosphatidic acid
HGSOC: high‐grade serous ovarian cancer
ER: endoplasmic reticulum
Taxol®: TX
Tg: thapsigargin
Mn^2+^
: manganese

## 1 Introduction

High‐grade serous ovarian cancer (HGSOC) is the most common subtype of epithelial ovarian cancer. Although referred to as ovarian cancer, the origin of the serous subtype was long debated. The histology of these tumors seems to be not strictly restricted to ovarian epithelium and it is now widely acknowledged that the majority originate from the epithelium of the fallopian tube (for review Lisio et al. [1]). In early stages of the pathology (stages I and II), HGSOC is localized on the ovary. However, the silent progression of HGSOC frequently leads to late detection and diagnosis once the cancer has spread to the peritoneal cavity (Stages III and IV) [1]. In the ascites and plasma of ovarian cancer patients, an increase in lysophosphatidic acid (LPA) level generally accompanies tumor development, and LPA was proposed as a diagnostic and prognostic marker for ovarian cancer [2, 3, 4]. *In vitro*, LPA, a bioactive phospholipid, enhances ovarian cancer cell migration and invasion [5, 6, 7]. HGSOC treatment combines a surgical procedure called ‘debulking surgery’ and taxane and platinum‐based chemotherapy [8, 9]. Unfortunately, ovarian cancer cells frequently develop drug resistance and the 5‐year survival rate is 20–30% [10, 11, 12].

Calcium‐activated potassium channels (KCa) are subdivided into three families based on their conductance: small (SKCa), intermediate (IKCa), and big (BKca). The SKCa subfamily is comprised of three members, SK1 (*KCNN1*), SK2 (*KCNN2*), and SK3 (*KCNN3*). These channels are formed by the association of four α‐subunits. These channel families have been demonstrated to be activated by increases in cytosolic Ca^2+^ concentration rather than through a voltage‐gated mechanism [13]. We previously showed that SK3 channel activity can hyperpolarize the plasma membrane, increasing the driving force for Ca^2+^ and potentiating the store‐operated Ca^2+^ entry (SOCE) and/or the constitutive Ca^2+^ entry (CCE) pathways and contributing to the migration of cancer cells [14, 15]. Synthetic lipids (Ohmline) can dissociate plasma membrane‐localized SK3 from the SOCE complex, leading to a decrease in cell migration in colon cancer cells [15].

In human cells, plasma membrane SKCa activity has been notably described in the regulation of neuron excitability [16]. SK2 channels were also identified in intracellular compartments, such as mitochondria, the endoplasmic reticulum (ER), or vesicles, and notably controlling cell death [17, 18, 19, 20]. Although there is little information on SKCa channels in cancers, these channels participated in cell proliferation and cell migration [21, 22, 23]. Our laboratory has described the role of plasma membrane SK3 in breast, melanoma, prostate, and colon cancer cell migration *in vitro* and in breast cancer metastasis *in vivo* [14, 15, 21, 24]. One recent article indicated that plasma membrane SK2 was responsible for pancreatic cancer cell migration and invasion [22] and involved in the proliferation of glioblastoma cells and in hypoxia‐induced proliferation of melanoma cells [25, 26]. To date, there is no data available on the role of SK2 channels in ovarian cancer cells and chemoresistance.

In this study, we explored the role of plasma membrane‐localized SK2 channels in HGSOC cell migration and chemoresistance. Additionally, we assessed the effects of LPA on SK2 channel activity in HGSOC cells.

## 2 Materials and methods

### 2.1 Cell culture and pharmacologic drugs

Human high‐grade serous ovarian cancer cell lines COV504 and OVCAR3 were maintained, respectively, in DMEM and RPMI 1640 medium (Gibco, Illkirch, France) supplemented with 10% fetal bovine serum (FBS, Hyclone Lonza Bioscience, Illkirch, France) under 5% CO_2_ at 37 °C. COV504 cells (RRID: CVCL_2424) were purchased from ECACC (Illkirch, UK) and OVCAR3 (RRID: CVCL_0465) cells were kindly provided by Dr Nadine Hempel's laboratory (University of Pittsburgh). Cells were received in 2020 and have been tested and authenticated by DNA 135 fingerprinting conducted by the ATCC (Manassas, VA, USA). Taxol®‐resistant sublines COV504 TX and OVCAR3 TX were generated after exposure to Taxol® (5 nm, Sigma‐Aldrich, Saint Quentin Fallavier, France) for 12 weeks. Then chemoresistant sublines were cultivated with Taxol® (5 nm) 3–4 days per week. The absence of mycoplasma contamination was regularly confirmed using the MycoAlert Mycoplasma Detection Kit (Lonza, Illkirch, France).

For SK2, the only current commercially available pharmacological inhibitor is Lei‐Dab7 is a cell impermeant peptide targeting plasma membrane channel (IC50: 3 nm, selectivity SK2>>>SK3>>SK1) [27]. Water‐soluble Lei‐Dab7 was used at 10 nm (Tocris, Bristol, UK). LPA was diluted in 0.4% bovine serum albumin before use at 10 μm (Aventi Polar Lipids, Alabaster, AL, USA).

### 2.2 Survival assay

Cell viability was analyzed using the MTT assay over a 7‐day period, as mentioned previously [28]. Cell lines were seeded in 48‐well plates (10 000 cells per well). Cells were then treated by a range of concentrations of Taxol® (0 to 160 nm) in their respective cell culture medium at 24 and 96 h after seeding. Cell survival rates in Taxol® conditions were normalized to control condition (without drug). An increase of EC_50_, the dose for which 50% of the drug's maximal effect is obtained, is associated to drug resistance.

### 2.3 Migration assay

Cells were seeded into migration inserts (8 μm pore size, in 24‐well plates, Falcon #353097, France), as described previously [14] and cell migration was induced by a 0–10% FBS gradient in the upper and lower compartment, respectively. Equal drug concentrations of Lei‐Dab7 (10 nm) were added in both compartments. After 24 h, an automatic count was performed on nuclei stained with DAPI (1 μg·mL^−1^, Sigma‐Aldrich) [29]. For a total of 50 000 cells seeded, we counted around 1325 migrated cells for COV504 and 1100 cells for OVCAR3.

### 2.4 Ca^2+^ measurements

Cytosolic Ca^2+^ was measured with the FlexStation3 (Molecular Devices, Wokingham, UK) at 37 °C using the Fura‐2‐AM probe (Invitrogen™, Illkirch, France) and fluorescence ratio method as mentioned previously [14, 15]. To increase the Fura‐2 signal, efflux pumps were inhibited with 2.5 mm of probenecid (Sigma‐Aldrich).

#### 2.4.1 Store‐operated Ca^2+^ entry

To measure store‐operated Ca^2+^ entry (SOCE), Ca^2+^ release from the endoplasmic reticulum was induced with Thapsigargin (4 μm, Invitrogen™) in PSS‐free‐Ca^2+^ solution. At 500 ms, the injection of 5 mm CaCl_2_, Ca^2+^ entry across the plasma membrane was measured as the maximum fluorescence ratio (F340/F380) minus the baseline value, which was normalized to the control condition.

#### 2.4.2 Constitutive Ca^2+^ entry measured by Mn^2+^ quenching method

Constitutive Ca^2+^ entry (CCE) was measured as described previously [30]. Fura‐2‐labeled cells were incubated with PSS Ca^2+^‐free solution, and 5 mm manganese (Mn^2+^) (Sigma‐Aldrich) was injected. The fluorescence emission was measured at 510 nm with excitation at 360 nm. The influx was analyzed by measuring the decreasing slope of fluorescence for 100 s after Mn^2+^ injection. The absolute value of the slope was normalized to the control condition.

### 2.5 Patch clamp experiments

Electrophysiological recordings of SKCa channel currents on COV504 were performed with the whole cell patch clamp technique, as described previously [14, 21, 31]. Data acquisition and analysis were performed using pclamp7 software (Axon Instruments, Union City, CA, USA). SK2 currents were generated by stepwise 10 mV depolarizing pulses (500 ms duration; 4 s intervals) from a constant holding potential of −90 mV up to +80 mV. To build IV relation, the average of steady state current at the end of the pulse (the last 50 ms) was chosen for each membrane potential.

### 2.6 RNA isolation and real‐time quantitative RT‐PCR

Total RNA extraction (NucleoSpin® RNA Plus kit, Macherey‐Nagel, Hoerdt, France), reverse transcription (PrimeScript RT kit, Takara, Saint Germain en Laye, France) and amplification (TB green Premix ExTaq kit, Takara) were performed as described previously [28]. The qPCR experiments were performed on CFX Maestro from Bio‐Rad (Marnes la Coquette, France) using 25 ng cDNA and the following specific primers: SK1(F) 5′‐CACCAAGGAGTCTCTGTACTC‐3′ and (R) 5′‐CAGTCATCAGCCCCGTTGT‐3′; SK2 (F) 5′‐GACTTGGCAAAGACCCAGAA‐3′ and (R) 5′‐CCGCTCAGCATTGTAAGTGA‐3′; SK3 (F) 5′‐TGGACACTCAGCTCACCAAG‐3′ and (R) 5′‐GTTCCATCTTGACGCTCCTC‐3′; HPRT1 (F) 5′‐TGACCTTGATTTATTTTGCATACC‐3′ and (R) 5′‐CGAGCAAGACGTTCAGTCCT‐3′; TBP (F) 5′‐TGTATCCACAGTGAATCTTGGTTG‐3′ and (R) 5′‐GGTTCGTGGCTCTCTTATCCTC‐3′; ALAS1 (F) 5′‐AGATCTGACCCCTCAGTCCC‐3′ and (R) 5′‐TCCACGAAGGTGATTGCTCC‐3′; h‐RNF‐181 (F) 5′‐GGGCCAGGAGCATAAGTACC‐3′ and (R) 5′‐ATCAGGAGGGCTCTCATCCA‐3′. Relative levels of transcripts (fold change, $Q=2^{-\Delta \Delta C_{T}}$) were calculated according to the ΔΔ*C*_T_ method against the median *C*_T_ of housekeeping genes (HPRT1, Alas‐1, TBP, and h‐RNF‐181).

### 2.7 Western blot

Proteins were extracted and separated as described previously [15, 30]. Proteins (50 μg) were separated with polyacrylamide gel electrophoresis (4–15% Mini‐PROTEAN® TGX Stain‐Free™ Protein Gels, Bio‐Rad) and transferred onto polyvinylidene fluoride membranes (Trans‐Blot Turbo Mini Transfer Packs, Bio‐Rad). After incubation with SK2 primary antibody (Clone K78/29 Merck, 1 : 1000, Saint Quentin Fallavier, France) and anti‐mouse antibody (1 : 3000 Jackson ImmunoResearch, West Grove, PA, US) coupled with horseradish peroxidase, luminescent signal was acquired using an ECL chemiluminescence kit (Clarity Western ECL substrate, Bio‐Rad) and ChemiDoc MP Imaging System (Bio‐Rad). Band quantification was performed on image lab software (Bio‐Rad) and total protein stain used for normalization.

### 2.8 Progression‐free survival analysis

We used the Kaplan–Meier plotter data base (kmplot.com) [32] to investigate the progression‐free survival between low and high *KCNN2* expression levels in serous ovarian cancer. Gene expression data and relapse‐free and progression‐free survival information are downloaded from GEO, EGA and TCGA. The database is handled by a PostgreSQL server, which integrates gene expression and clinical data simultaneously. To analyze the prognostic value of a particular gene, the patient samples are split into two groups according to various quantile expressions of the proposed biomarker (auto select best cutoff). The two patient cohorts are compared by a Kaplan–Meier survival plot, and the hazard ratio with 95% confidence intervals and logrank *P* value are calculated. The best‐performing threshold was used as a cutoff. We selected patients by type of treatment (Taxol® or platin) and stages of the disease: early (stages I and II) and late (stages III and IV) [33].

### 2.9 Immunofluorescence

Approximately 5000 cells were seeded in presence or absence of LPA (10 μm) on Lab‐Tek II chamber slides(ref 154534 Fisher Scientific, Illkirch, France) 72 h prior to the experiment. After washing with PBS, cells were fixed with PFA (4%) at room temperature for 15 min. Thereafter, cells were washed and permeabilized with Triton™ X‐100 (0.1% in PBS; Sigma) and blocked with BSA 3% for 30 min. Primary antibody (IHC‐plus KCNN2 antibody, Clinisciences, Nanterre, France) was diluted at 1 : 500 and incubated overnight at 4 °C. Secondary antibody was added at 1 : 1000 at room temperature for 1 h (Goat anti‐rabbit Alexa488, AbCam, Cambridge, UK). Fluorescence was visualized using High Resolution Laser Scanning Leica SP8 gSTED Confocal Microscope (Leica) and las‐x v2.0 software (Leica, Wetzlar, Germany). Images were analyzed using fiji (imagej software, National Institutes of Health, US).

### 2.10 siRNA transfection

Cells were transfected with small interfering RNA (siRNA) using a Lipofectamine RNAiMax transfection reagent (Invitrogen, France). We used the following siRNA sequences (Eurogentec, Seraing, Belgium) 5′GACAAGCACGUCACUUACA3′ (siSK2) and, 5′CUGUAUCGAAUGUUAUGAGCC3′ (control siRNA). Transfections were performed with 1 : 1.5 siRNA/Lipofectamine ratio and 20 nm siRNA concentration.

### 2.11 Statistics

The results from the calcium measurement, mRNA expression levels and migration assays were normalized to the control condition and represented as mean ± SEM. The efficacy dose EC_50_ from survival assays, western blot and patch clamp experiments were analyzed as non‐parametric and paired data (Wilcoxon test). Statistical significance was established for *P* < 0.05*, *P* < 0.01**, *P* < 0.001***, using graphpad 8.01 software (Boston, MD, USA).

## 3 Results

### 3.1 SK2 channels contribute in SOCE and migration in HGSOC cells

**Fig. 1:**
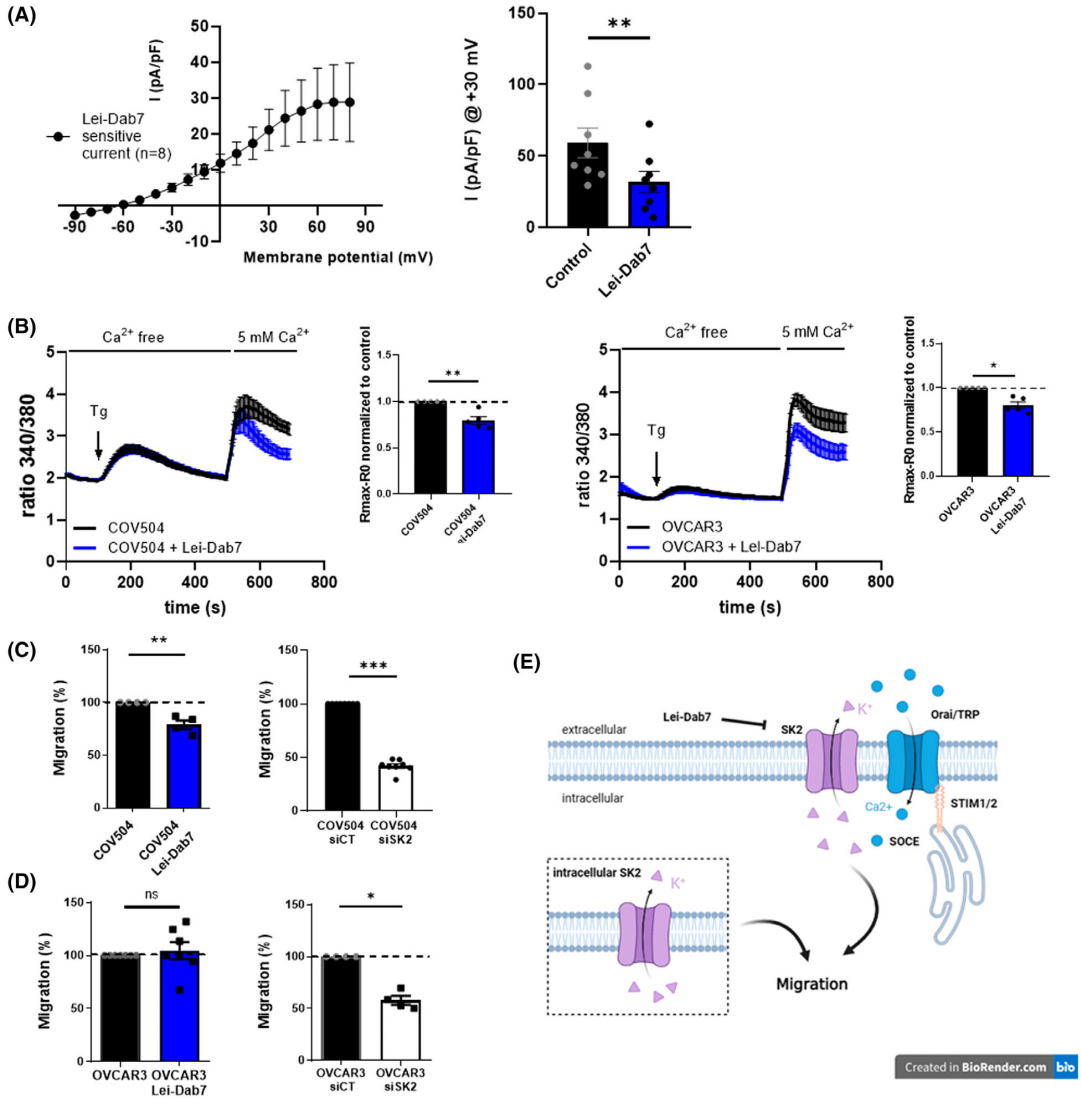
SK2 channels contribute in store‐operated calcium entry (SOCE) and migration in high‐grade serous ovarian cancer (HGSOC) cells. (A) Effect of Lei‐Dab‐7 on current–density amplitude of COV504 cells. Lei‐Dab7 sensitive current–density–voltage relationships during voltage step cells before and after Lei‐Dab7 (10 nm) perfusion (*N* = 8). Sensitive current was obtained by subtracting the outward current recorded in the presence of Lei‐Dab7 from the net outward current observed in normal physiological saline solution (left panel). Current density measured at +30 mV (right panel). (B) SOCE measurements in COV504 (left panel) (*n* = 12, *N* = 5) or OVCAR3 cells (right panel) (*n* = 17, *N* = 5) using Fura‐2‐AM with or without acute Lei‐Dab7 administration. (C and D) Transwell migration assay with cells treated or not with Lei‐Dab7 (left panels) (COV504, N4; OVCAR3, *N* = 7) or siSK2 (right panels) (COV504, *N* = 8; OVCAR3, *N* = 4). (E) Plasma membrane and intracellular SK2 channel pools that contribute to cell migration and SOCE in HGSOC. The cell migration (C and D) and the Ca^2+^ measurement amplitudes (B) were expressed as mean ± SEM, each point is the result of one experiment, and is normalized to the control condition (Mann–Whitney, *P* < 0.05*, *P* < 0.01**, *P* < 0.001***). The whole cell recording results (A) are represented as mean ± SEM (Wilcoxon, *P* < 0.01**) (Tg, Thapsigargin).

We used whole cell recordings and Lei‐Dab7 (10 nm) to identify a SK2 current at the plasma membrane of COV504 cells (Fig. S1A). We unmasked the Lei‐Dab7 sensitive current with an inward rectification, characteristic of SKCa channels (Fig. 1A). Acute application of Lei‐Dab7 on COV504 cells significantly decreased the global current from 59.13 ± 10.48 to 31.82 ± 7.41 pA per pF at +30 mV (Fig. 1A). Combined with immunofluorescence analysis of SK2 expression (Fig. S2C), all these results supported the idea that SK2 channels were functional and localized at the plasma membrane of COV504 cells. Acute Lei‐Dab7 treatment was responsible for a significant decrease in SOCE (~ 20%) in both COV504 and OVCAR3 cells (Fig. 1B). No difference in the constitutive Ca^2+^ entry after Lei‐Dab7 treatment was detected (Fig. S1B). Lei‐Dab7 significantly decreased the migration of COV504 cells by ~ 20% (Fig. 1C). With siRNA targeting *KCNN2* (siRNA selectivity, Fig. S1C), the effect was more striking, with a ~60% decrease in cell migration (Fig. 1C). In OVCAR3 cells, Lei‐Dab7 did not alter cell migration, while SK2 silencing significantly decreased the migration by ~ 50% (Fig. 1D). Furthermore, the activation of SKCa channels by CyPPA significantly increased migration of COV504 but not OVCAR3 cells (Fig. S1D).

Taken together, our results showed that functional SK2 channels were localized at the plasma membrane (Fig. S2C) and involved in SOCE in both COV504 and OVCAR3 cells. In COV504 cells, functional plasma membrane SK2 channels were involved in cell migration. SK2 silencing experiments in both COV504 and OVCAR3 cells suggest the involvement of an intracellular pool of SK2 in cell migration (Fig. 1E).

### 3.2 LPA increases the activity of plasma membrane SK2 channels and cell migration in a SOCE‐independent manner

**Fig. 2:**
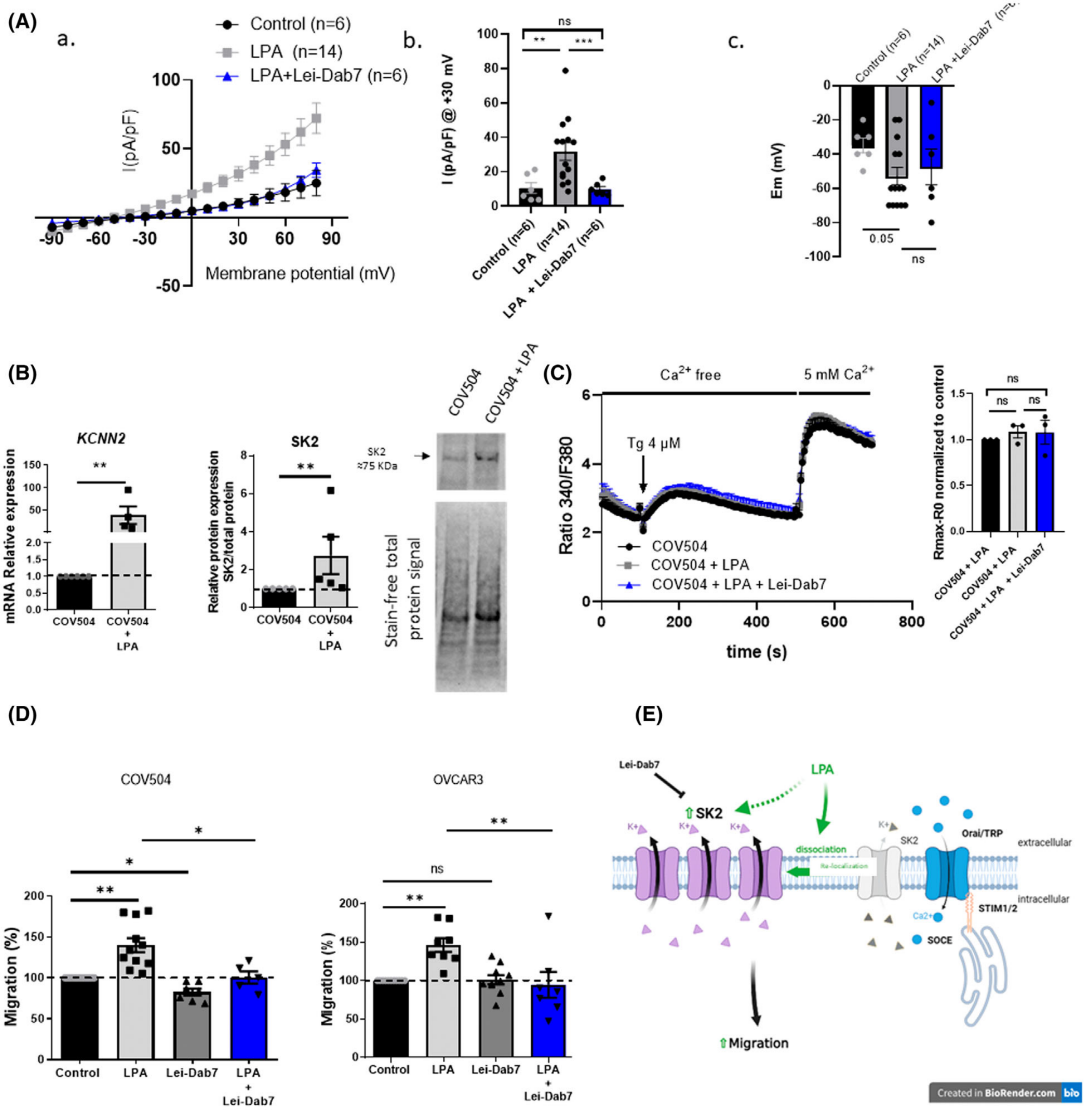
LPA increases the activity of plasma membrane SK2 and cell migration in a store‐operated calcium entry (SOCE) independent manner. (A) Effect of LPA pre‐treatment on SK2 current. Current density–voltage relationships obtained on COV504 cells before and after LPA treatment (10 μm) for 72 h and with or without acute Lei‐Dab7 application (10 nm) (PSS Control, *N* = 6; LPA, *N* = 14; LPA + Lei‐Dab7, *N* = 6) (A). Current‐density measured at +30 mV (B) and evaluation of membrane potential (Em) on COV504 cells control and pre‐treated with LPA ± Lei‐Dab7 (C). (B) *KCNN2* mRNA (left panel) and SK2 protein (right panel) expression level quantified by qRT‐PCR (*N* = 4) and western blot (*N* = 4), respectively. Measurements were performed on COV504 cells with or without LPA pre‐treatment (10 μm). (C) SOCE measurements in COV504 cells (*n* = 16, *N* = 4) using Fura‐2‐AM fluorescent probe with or without acute Lei‐Dab7 application (10 nm) on cells, in the presence or absence of LPA for 72 h (10 μm). (D) Transwell migration assay with COV504 (left panel) (*N* = 5) or OVCAR3 (right panel) (*N* = 7) cells with or without Lei‐Dab7 (10 nm) or LPA pre‐treatment (72 h, 10 μm) for 24 h assay. (E) Hypothetical representation of LPA activity that induces SK2‐dependent cell migration but loss of SK2 channel participation in SOCE in HGSOC cells. Whole cell recording (A) is expressed as mean ± SEM (Wilcoxon, *P* < 0.05*, *P* < 0.01**, *P* < 0.001***). Results from qRT‐PCR (B, left panel), western blots (B, right panel), Ca^2+^ measurements (C), and migration assay (D) are expressed as mean ± SEM, each point is the result of one experiment, and is normalized to the control condition (Mann–Whitney, *P* < 0.05*, *P* < 0.01** (B), One‐way ANOVA, *P* < 0.05*, *P* < 0.01** (C, D)) (LPA, lysophosphatidic acid; Tg, Thapsigargin).

Cells were exposed to 10 μm of lysophosphatidic acid (LPA) for 72 h. In COV504 cells, we noticed a significant increase in outward current (Fig. 2A, a) from 10.55 ± 3.16 to 31.79 ± 5.09 pA per pF at +30 mV (Fig. 2A,B), and acute injection of Lei‐Dab7 abolished LPA‐induced outward current from 31.79 ± 5.09 to 11.07 ± 1.91 pA per pF at +30 mV. LPA tended to hyperpolarize the membrane potential (*P* = 0.05) and Lei‐Dab7 application did not alter it (Fig. 2A,C). These data show for the first time that SK2 channel activity is regulated by LPA. In COV504, LPA pre‐treatment increased *KCNN2* mRNA by 35 fold and SK2 protein level by 2.7 fold (Fig. 2B). In OVCAR3, LPA enhanced *KCNN2* mRNA expression levels by 2 fold but did not affect SK2 protein levels (Fig. S2A). The expression level of *KCNN1* and *KCNN3* after LPA treatment is shown in Fig. S3A. Despite of *KCNN2* mRNA and SK2 level changes, we surprisingly observed no increase in SOCE activity, and SOCE sensitivity to Lei‐Dab7 was lost after LPA treatment in both cell lines (Fig. 2C and Fig. S2B). These results suggest that SK2 no longer participates to SOCE in LPA‐treated cells.

As expected, and previously described in literature, LPA pre‐treatment significantly increased cell migration by ~ 40% in COV504 and ~ 50% in OVCAR3 (Fig. 2D). SK2 inhibition by Lei‐Dab7 abolished LPA‐induced cell migration in both cell lines (Fig. 2D). Our data show for the first time that LPA strongly induces SK2 activity at the plasma membrane and LPA‐induced cell migration is totally suppressed by inhibition of SK2 activity in both COV504 and OVCAR3, demonstrating that SK2 is a key regulator of LPA pro‐migratory effect. As shown by immunofluorescence analysis, SK2 localization, in both cells lines, seems to be modified after LPA treatment (Fig. S2C). Furthermore, we showed that the LPA‐treated cell migration was SOCE‐independent, as the pharmacological inhibition of SOCE by synta66 did not modified LPA‐treated cell migration (Fig. S3B). These results suggest that LPA altered the membrane localization of SK2 and its association with SOCE channel complexes (Fig. 2E).

### 3.3 Plasma membrane SK2 channel activity increases sensitivity to chemotherapy

**Fig. 3:**
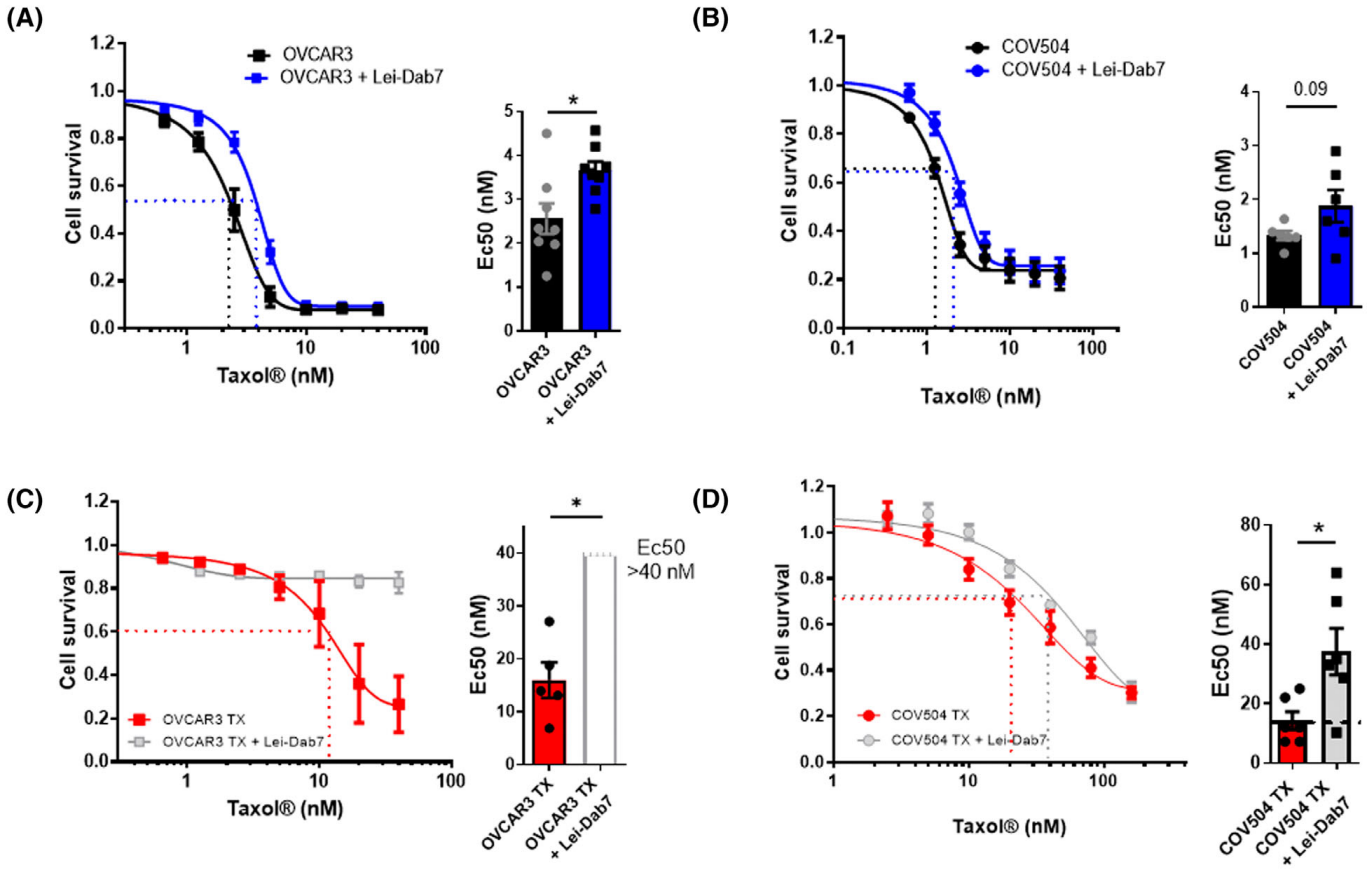
Plasma membrane SK2 activity increases Taxol® sensitivity in sensitive and resistant cell lines. In the presence or absence of Lei‐Dab7 (10 nm), cells were treated with a range of Taxol® concentration to evaluate EC_50_ values in a 7‐day survival assay in OVCAR3 (*N* = 8) (A), and COV504 (*N* = 6) (B), OVCAR3 TX (*N* = 5) (C), and COV504 TX (*N* = 6) (D) cells. EC_50_ is the dose for which we obtained 50% of the drug's maximal effect. EC_50_ increase is associated with drug resistance. On the right, histograms represent EC_50_ values in the presence of Lei‐Dab7. Each point is the result of one experiment. For the statistical analysis of EC_50_ in OVCAR3 TX + Lei‐Dab7 condition, the maximal value of the Taxol® range was used (40 nm) (representing by a dot line on C right panel). The survival assay results are expressed as mean ± SEM (Wilcoxon, *P* < 0.05*) (OVCAR3 TX and COV504 TX = Taxol® resistant cells).

We investigated the role of SK2 in Taxol® resistance by using cell survival assays for 7 days. Inhibition of SK2 channel activity by Lei‐Dab7 significantly increased EC_50_ by ~ 60% in OVCAR3 cells (Fig. 3A). In COV504 cells (Fig. 3B), EC_50_ tended to increase by ~ 43% (*P* = 0.094).

Then, we generated Taxol®‐resistant sublines, COV504 TX and OVCAR3 TX, derived from COV504 and OVCAR3 cells. EC_50_ was increased by 6 fold and 1.5 fold in COV504 TX and OVCAR3 TX, respectively (Fig. S4A). In COV504 TX, KCNN2 mRNA expression level increased by 7 fold and SK2 protein level increased by 1.5 fold (Fig. S4B). In OVCAR3 TX, KCNN2 mRNA expression level increased by 4 fold but SK2 protein level was unchanged (Fig. S4C) Similarly to sensitive cell lines (Fig. 1B), we confirmed that Lei‐Dab7 significantly reduced SOCE in the resistant sublines respectively by ~ 15% and ~ 30% in COV504 TX and OVCAR3 TX cells (Fig. S4D). These results showed SK2 channels remained associated to SOCE complex in resistant cell lines.

Inhibition of SK2 activity by Lei‐Dab7 increased Taxol® resistance in both COV504 TX and OVCAR3 TX (Fig. 3). In OVCAR3 TX cells, the resistance developed by Lei‐Dab7 administration was so large that the cell viability decrease did not appear with the range concentration of Taxol® we used (EC_50_ greater than 40 nm) (Fig. 3C). In COV504 TX, the EC_50_ value increased by 2.8 fold after Lei‐Dab7 treatment (Fig. 3D). In both resistant and sensitive cell lines, Lei‐Dab7 at 10 nm did not affect the cell viability measured in the same conditions after a 7‐day culture (Fig. S4E). These data provided the first evidence that plasma membrane SK2 activity increased Taxol® sensitivity in HGSOC cells.

To date, there is no selective activator of plasma membrane SK2 channels, but we tested a non‐selective and cell permeant SKCa channel activator, CyPPA (selectivity: SK3>SK2>>>SK1). In both parental cell lines, CyPPA (1 μm) decreased the EC_50_ by ~ 35% in COV504 and by ~ 50% in OVCAR3, suggesting an increase in Taxol® sensitivity. In Taxol®‐resistant cells, CyPPA induced a dramatic cytotoxic effect of CyPPA was observed (Fig. S5A,B).

**Fig. 4:**
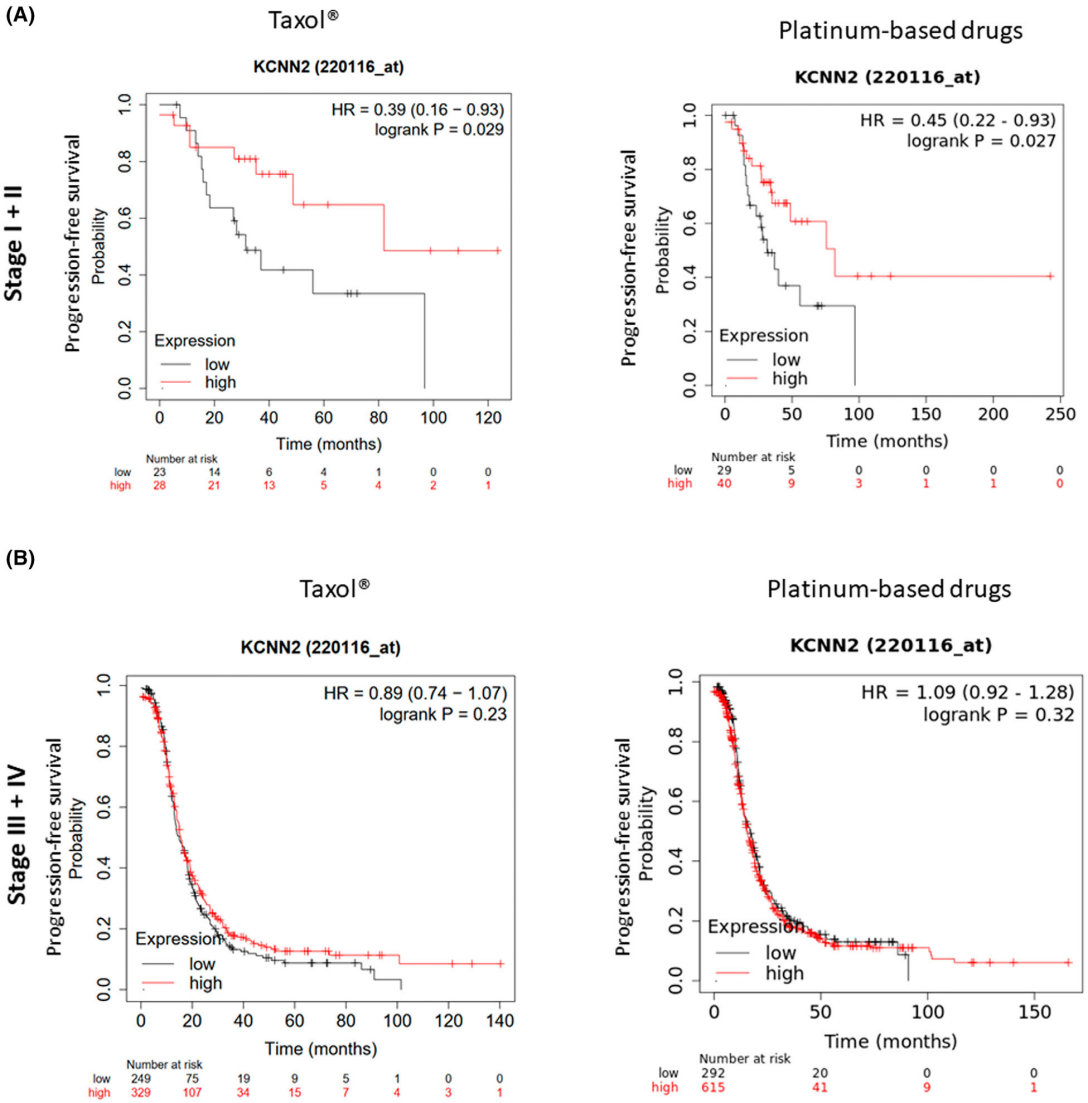
High *KCNN2* mRNA expression was associated with better survival rates among patients with early stages of serous ovarian cancer. (A) Progression‐free survival of patients with stages I and II of serous ovarian cancer depending on *KCNN2* expression level. Kaplan–Meier curves represented patients treated with Taxol® (left panel, *P* value 0.0289, FDR over 50%) or platinum‐based drugs (right panel, *P* value 0.0266; FDR over 50%) (Taxol®, *N* = 51; platinum‐based drugs, *N* = 69). (B). Progression‐free survival of patients with stages III and IV of serous ovarian cancer treated with Taxol® (left panel, *P* value 0.2269, FDR 100%) or platinum‐based drugs (right panel, *P* value 0.3228, FDR 100%) (Taxol®, *N* = 578; platinum‐based drugs, *N* = 907) depending on *KCNN2* expression levels (kmplot.com).

To explore the clinical relevance of *KCNN2* expression level on the progression‐free survival of patients with serous ovarian cancer, we used the Kaplan–Meier plotter database. We noticed that in the early stages (I and II), patients treated with Taxol® or platinum‐based drugs received better prognoses when high *KCNN2* levels were present in the tumors (Fig. 4A). However, *KCNN2* mRNA level was not related to the progression‐free survival of patients with late stages (III and IV) who underwent either of the chemotherapies (Fig. 4B).

Thus, high *KCNN2* mRNA level could be associated with a better outcome from chemotherapy treatment in the early stages of serous ovarian cancer, but the benefits were lost in more advanced forms of the disease.

## 4 Discussion

Our results show that plasma membrane‐localized SK2 participates to SOCE in HGSOC cells and plays a key role in LPA‐induced cell migration in a SOCE independent manner. Furthermore, we discovered that plasma membrane SK2 inhibition increased chemoresistance to Taxol® and that *KCNN2* mRNA expression level may constitute a prognostic marker for early stages of serous ovarian cancer.

### 4.1 Plasma membrane and intracellular SK2 regulate cell migration

A differential effect between SK2 knockdown and the SK2 peptide inhibitor Lei‐Dab7 (Fig. 1) was observed on cell migration. These data would suggest the existence of two pools of SK2 channels: intracellular pool and at the plasma membrane‐localized pool. It is known that SKCa channels localized at the plasma membrane control neural excitability, cell migration *in vitro* and *in vivo* cancer metastasis [14, 16, 34]. However, functional SKCa channels, in particular the SK2 and SK3 subtypes, were identified at the inner mitochondrial membrane of neuronal and cardiac cells and protected cells against death [35, 36, 37]. Functional SK2 in endoplasmic reticulum (ER) unmasked by CyPPA (a cell permeant and potent activator of SKCa channels) was also observed to be protective against ER stress‐induced cell death [19]. Our SOCE assays suggest that SK2 channels can contribute to SOCE but this contribution is lost under LPA treatment. In neurons and cardiomyocytes, plasma membrane SK2 localization is dependent upon a large variety of partners, such as palmitoylated protein 2, alpha‐actin‐binding protein 2, or filamin A, which enable its trafficking to the plasma membrane through endosomes [18, 20]. Moreover, it has been reported that SK3 activity depends on trafficking by caveolae, regulating its localization and possibly the number of channels at the plasma membrane [38, 39]. LPA may relocate SK2 channels Altering the activity of cytoskeleton proteins [6] and thereby disrupt the regulation of vesicular transport.

Previously, we described the involvement of plasma membrane SK3 channels in SOCE and constitutive Ca^2+^ entry and found that SK3 was localized within nanodomains of the plasma membrane [15, 21, 34]. The association of SK2 channels with SOCE was suggested to be restricted to specific nanodomains of the plasma membrane [40] but this remained to be demonstrated in our cells. Nevertheless, the absence of a shift in the membrane potential after Lei‐Dab7 treatment may suggest that SK2 potentiates SOCE by promoting a local hyperpolarization to enhance the driving force for Ca^2+^. However, current clamp experiments should be carried out to measure the resting membrane potential more accurately.

Under LPA treatment SK2 channels no longer participate to SOCE. Here, we cannot exclude that LPA may promote the phosphorylation of SK2 channels or their partners to provoke their plasma membrane delocalization. This phenomenon (phosphorylation/dissociation) was previously described for the ORAI/SK3 channel complex [40]. It is also known that lipid environments can alter the curvature of the plasma membrane, delocalize channels, and promote their activation [34]. Notably, LPA can activate TREK channels (2‐pore domain K^+^ channel) using a similar mechanism [41, 42]. From this perspective, a direct LPA insertion into the plasma membrane could lead to SK2 delocalization. Nevertheless, LPA interaction with its receptors up‐regulated SK2 transcriptionally, as shown in COV504 cells. Additionally, LPA may also recruit intracellular SK2, originally trapped in endosomes and mobilized by cytoskeletal protein activation [6, 20]. Finally, LPA can promote ovarian cancer cell migration by activating an epidermal growth factor receptor (EGFR)‐dependent pathway. Indeed, it was recently reported that SK2 activity promoted cell migration and invasion in pancreatic cancer cells by enhancing an integrin–EGFR–AKT (Protein kinase B) pathway [22, 43].

### 4.2 SK2 contribution to chemosensitivity

Plasma membrane SK2 inhibition increased resistance to Taxol® in HGSOC cell lines. Several studies reported inhibition or downregulation of K^+^ channel led to chemoresistance in cancers [44]. In the literature, a prominent hypothesis is that the increase of K^+^ efflux and/or reduction of intracellular K^+^ concentration can lead to the activation of pro‐apoptotic factors, such as interleukin‐1β (IL‐1β), reactive oxygen species, or Caspase 1, thus promoting sensitivity to chemotherapies. In addition, promoting membrane hyperpolarization, the K^+^ efflux can potentiate a cytosolic Ca^2+^ increase, and contribute to the induction of mitochondrial Ca^2+^ overload and cell death [45]. For KCa specifically, their expression and activity potentiate cisplatin drug uptake into the cell leading to chemosensitivity. Unfortunately, the related mechanism of action through which KCa channels mediate this effect is still unclear [46].

In a concordant manner, the KM‐plotter analysis showed that higher *KCNN2* expression levels are associated with a better prognosis for patients treated with Taxol® or platinum‐based drugs. However, while this phenomenon was observed for early stages, it was not observed for late stages of serous ovarian cancer. These results agreed with analysis performed on sarcoma tumors [47]. In addition, higher *KCNN3* mRNA expression levels were associated with a better prognosis in patients with ovarian cancer [48]. Thus, mRNA levels of *KCNN2* and *KCNN3* channels may constitute a promising prognostic marker or interesting targets to regulate chemosensitivity of several cancers.

Overall, both facets of SK2 could be exploited in the course of patient care. SK2 activators could be used as adjuvants to chemotherapy to enhance its efficacy, while SK2 inhibitors could be administered as monotherapy to limit the spread of cancer cells.

## 5 Conclusion

Our results showed for the first time that SK2 is involved in ovarian cancer cell migration and that plasma membrane SK2 plays a key function in LPA‐induced cell migration, suggesting SK2 as potential target that should be inhibited in context of the tertiary prevention after the end of chemotherapy cycles. Additionally, SK2 activity increases chemotherapy efficacy and *KCNN2* expression levels may constitute a prognostic marker for early stages of serous ovarian cancer. However, the mRNA levels of *KCNN2* were not sufficient to predict SK2 activity, cellular localization and function. Thus, while plasma membrane SK2 should be blocked to control cancer cell migration, its activity should be potentiate during chemotherapy to sensitize ovarian cancer cells. The activation or inhibition of SK2 should be adapted according to the stages of the patient's care pathway. Currently available tools to modulate SKCa channels are rare and lack selectivity and their development could provide a useful strategy to improve ovarian cancer outcomes.

## Conflict of interest

Mohamed Trebak is a paid consultant of Seeker Biologics Inc. The other authors declare no conflict of interest.

## Author contributions

OR wrote the manuscript that was critically reviewed by MP‐C, CG, NH and MT. OR, AL, OC, AC, and NC performed the experiments. LO provided clinical expertise. NH provided cell lines. MP‐C, CG and MT supervised the project.

### Peer review

The peer review history for this article is available at https://www.webofscience.com/api/gateway/wos/peer‐review/10.1002/1878‐0261.13631.

## Supporting information


**Fig. S1.** Functional plasma membrane SK2 does not participate in the CCE, and siSK2 specifically decreases *KCNN2* but not *KCNN1* or *KCNN3*.
**Fig. S2.** In OVCAR3, LPA treatment increases *KCNN2* mRNA levels without major impact on protein level and promotes the loss of sensitivity of SOCE to Lei‐Dab7.
**Fig. S3.** Effect of LPA treatment on expression of *KCNN1*, *KCNN2* and *KCNN3* in COV504 and OVCAR3 cells and effect of SOCE inhibition on LPA‐treated cell migration.
**Fig. S4.** Characterization of Taxol® chemoresistant sublines and effects of Lei‐Dab7 on SOCE and cell viability.
**Fig. S5.** CyPPA, a SK2 and SK3 activator significantly decreases Taxol® resistance in COV504 and OVCAR3 cells.

## Data Availability

The data that support the findings of this study are available from the corresponding author (marie.potier-cartereau@univ-tours.fr) upon reasonable request.
