# Supplementary material for: Plasma membrane SK2 channel activity regulates migration and chemosensitivity of high‐grade serous ovarian cancer cells
Source: Mol Oncol. 2024 Mar 13;18(8):1853–65. doi: 10.1002/1878-0261.13631 (PMC11306528; doi:10.1002/1878-0261.13631)
Supplement: Supplementary file 1 — Fig. S1. Functional plasma membrane SK2 does not participate in the CCE, and siSK2 specifically decreases KCNN2 but not KCNN1 or KCNN3. Fig. S2. In OVCAR3, LPA treatment increases KCNN2 mRNA levels without major impact on protein level and promotes the loss of sensitivity of SOCE to Lei‐Dab7. Fig. S3. Effect of LPA treatment on expression of KCNN1, KCNN2 and KCNN3 in COV504 and OVCAR3 cells and effect of SOCE inhibition on LPA‐treated cell migration. Fig. S4. Characterization of Taxol® chemoresistant sublines and effects of Lei‐Dab7 on SOCE and cell viability. Fig. S5. CyPPA, a SK2 and SK3 activator significantly decreases Taxol® resistance in COV504 and OVCAR3 cells. [file MOL2-18-1853-s001.zip › Supplementary figure legends.docx]

**Supplementary figure legends**

**Figure S1: Functional plasma membrane SK2 does not participate in the CCE, and siSK2 specifically decreases *KCNN2* but not *KCNN1* or *KCNN3*. A.** Effect of Lei-Dab7 on current density amplitude of COV504 cells. Current density-voltage relationships obtained on COV504 cells before and after acute application of Lei-Dab7 (10 nM) (N = 8). **B.** Mn^2+^ quench measurements performed in COV504 (**left panel**) (n = 20, N = 7) or OVCAR3 (**right panel**) (n = 18, N = 7) cells, treated or not with Lei-Dab7. The absolute value of the slope after Mn^2+^ injection represents the CCE. **C.** Specificity of siRNA SK2 (20 nM) measured by qRT-PCR (N = 4). The Ca^2+^ measurements (B) and qRT-PCR results (C) are expressed as mean ± SEM, each point is the result of one experiment, and is normalized to the control condition (Mann-Whitney, p<0.05*, p<0.01**, p<0.001***); (CCE = constitutive calcium entry). **D**. Transwell migration assay with COV504 (left panel) (N = 8) or OVCAR3 (right panel) (N=5) cells with or without CyPPA (1µM) for 24 h assay. Results are expressed as mean ± SEM, each point is the result of one experiment, and is normalized to the control condition, without cyppa (Mann-Whitney, p<0.05*)

**Figure S2: Figure S2: In OVCAR3, LPA treatment increases *KCNN2* mRNA levels without major impact on protein level, and promotes the loss of sensitivity of SOCE to Lei-Dab7. A.** *KCNN2* mRNA (**left panel**) (N = 5) and protein (**right panel**) (N = 3) expression level measured in OVCAR3 cells with or without LPA-pre-treatment (10 µM for 72h). **B.** SOCE measurements in OVCAR3 cells (n = 15, N= 4) using Fura-2-AM with or without acute Lei-Dab7 (10 nM) administration in cells with and without LPA pre-treatment (10 µM for 72h). The qRT-PCR, western blot, and Ca^2+^ measurements were expressed as mean ± SEM and are normalized to the control condition. Each point represents one experiment (Mann-Whitney, p<0.05*, p<0.01**, p<0.001***). C. Immunofluorescence labeling of SK2 channels in COV504 cells (left panel) and in OVACR3 cells (right panel) in presence or absence of LPA (10µM for 72h). Confocal microscope images showing re-distribution of SK2 channel after LPA treatment. (LPA = Lysophosphatidic acid; SOCE = store operated calcium entry, Tg = Thapsigargin).

**Figure S3: Effect of LPA treatment on expression of *KCNN1*, *KCNN2* and *KCNN3* in COV504 and OVCAR3 cells and effect of SOCE inhibition on LPA-treated cell migration.** **A.** *KCNN1, KCNN2* and *KCNN3* mRNA expression measured in COV504 (left panel) (N = 5) or in OVCAR3 (right panel) (N = 5) cells treated by LPA (10 µM for 72h) relative to untreated cells. Results are dispayed as mean ± SEM. Each point represents one experiment (Mann-Whitney, p<0.05*, p<0.01**, p<0.001***). **B**. Transwell migration assay with COV504 (left panel) (N = 5) or OVCAR3 (right panel) (N=3) cells with or without Synta66 (2 µM), an inhibitor of SOCE or Lei-Dab7, SK2 inhibitor (10 nM). Results are expressed as mean ± SEM, each point is the result of one experiment, and is normalized to the control condition (Mann-Whitney, p<0.05*); (LPA = Lysophosphatidic acid; SOCE = store operated calcium entry, Tg = Thapsigargin).

**Figure S4: Caracterization of Taxol® chemoresistant sublines and effects of Lei-Dab7 on SOCE and cell viability. A.** Cell survival assay in COV504 and COV504 TX (N = 8) (**left panel**) or in OVCAR3 and OVCAR3 TX cells (N = 5) (**right panel**) treated with a range concentration of Taxol®. EC_50_ is the dose for which we obtained 50% of the drug’s maximal effect. EC_50_ increase is associated with drug resistance. Histograms represent EC_50_ values (**right panel**). **B**. Expression of *KCNN2* mRNA (left panel) (N = 7) and SK2 protein (right panel) (N = 10) measured in COV504 TX relative to COV504 cells. **C**. Expression of *KCNN2* mRNA (left panel) (N = 4) and SK2 protein (right panel) (N = 4) measured in OVCAR3 TX relative to OVCAR3 cells. **D**. SOCE measurements in COV504 TX (left panel) (n = 15, N = 4) or OVCAR3 TX cells (right panel) (n = 17, N = 6) using Fura-2-AM with or without acute Lei-Dab7 administration. **E**. Cell viability of COV504 (N = 7) and COV504 TX (N = 3) (left panel) and OVCAR3 (N = 6) and OVCAR3 TX (N = 6) (right panel) treated or not with Lei-Dab7 (10 nM) for seven days. The survival assay results are expressed as mean ± SEM, each point represents one experiment (Wilcoxon, p<0.05*, p<0.01**, p<0.001***). The cell viability and Ca2+ measurements are normalized to the control condition expressed as mean ± SEM (Mann-Whitney, p<0.05*, p<0.01**, p<0.001***); (LPA = Lysophosphatidic acid; SOCE = store operated calcium entry, Tg = Thapsigargin; COV504 TX and OVACR3 TX = Taxol® resistant cells).

**Figure S5: CyPPA, a SK2 and SK3 activator significantly decreases Taxol® resistance in COV504 and OVCAR3 cells.**. **A and B**. Cell survival assay in COV504 **(B**) (N = 6) or in OVCAR3 (**C**) (N = 8) cells treated with a range concentration of Taxol® concentration with or without 1 µM CyPPA. Histograms represent EC_50_ values. The survival assay results are expressed as mean ± SEM, each point represents one experiment (Wilcoxon, p<0.05*, p<0.01**, p<0.001***).
