# Supplementary figures and images for: Plasma membrane SK2 channel activity regulates migration and chemosensitivity of high‐grade serous ovarian cancer cells
Source: Mol Oncol. 2024 Mar 13;18(8):1853–65. doi: 10.1002/1878-0261.13631 (PMC11306528; doi:10.1002/1878-0261.13631)

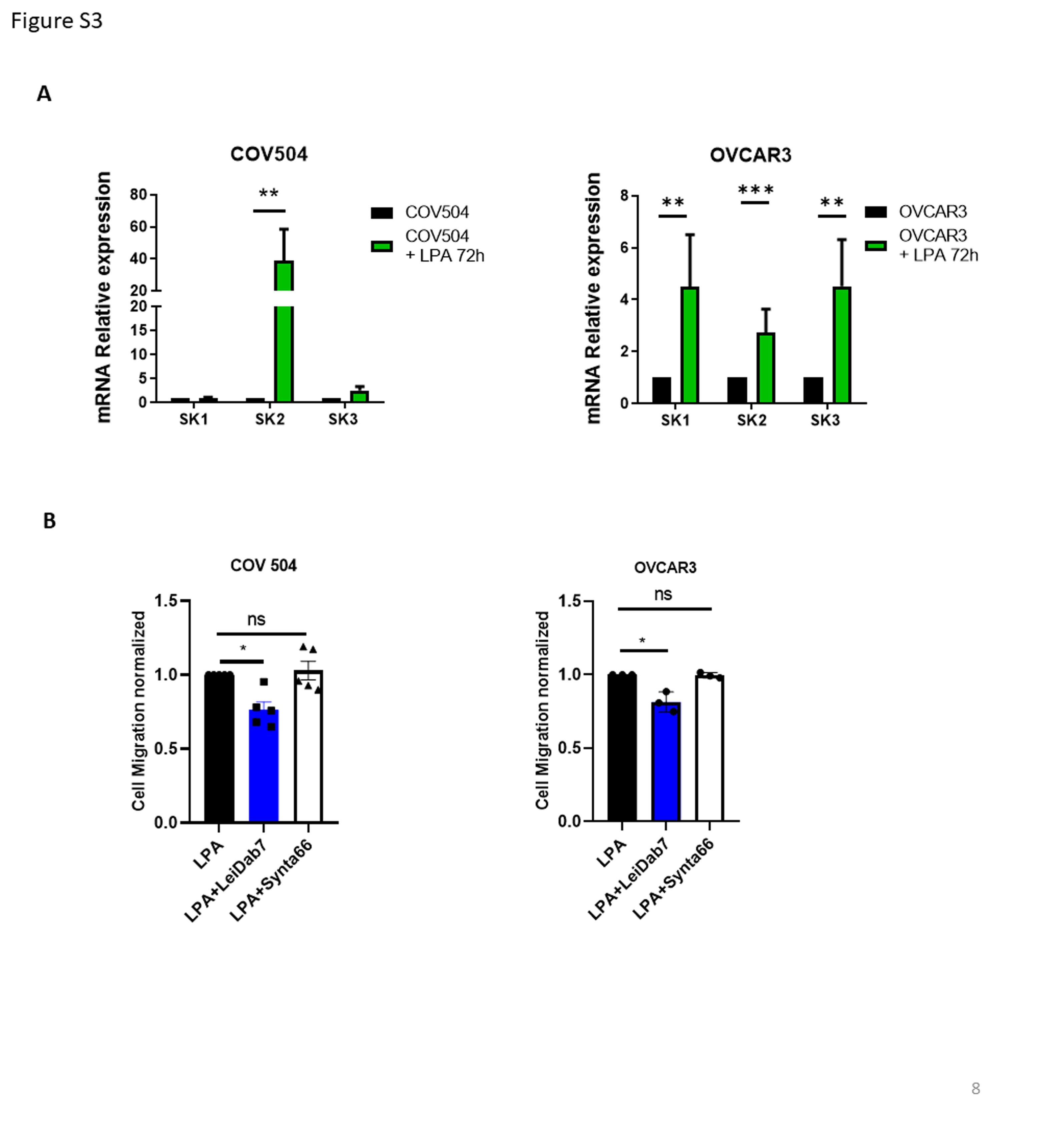

Supplement: Supplementary file 1 — Fig. S1. Functional plasma membrane SK2 does not participate in the CCE, and siSK2 specifically decreases KCNN2 but not KCNN1 or KCNN3. Fig. S2. In OVCAR3, LPA treatment increases KCNN2 mRNA levels without major impact on protein level and promotes the loss of sensitivity of SOCE to Lei‐Dab7. Fig. S3. Effect of LPA treatment on expression of KCNN1, KCNN2 and KCNN3 in COV504 and OVCAR3 cells and effect of SOCE inhibition on LPA‐treated cell migration. Fig. S4. Characterization of Taxol® chemoresistant sublines and effects of Lei‐Dab7 on SOCE and cell viability. Fig. S5. CyPPA, a SK2 and SK3 activator significantly decreases Taxol® resistance in COV504 and OVCAR3 cells. [file MOL2-18-1853-s001.zip › figure S3.TIF]

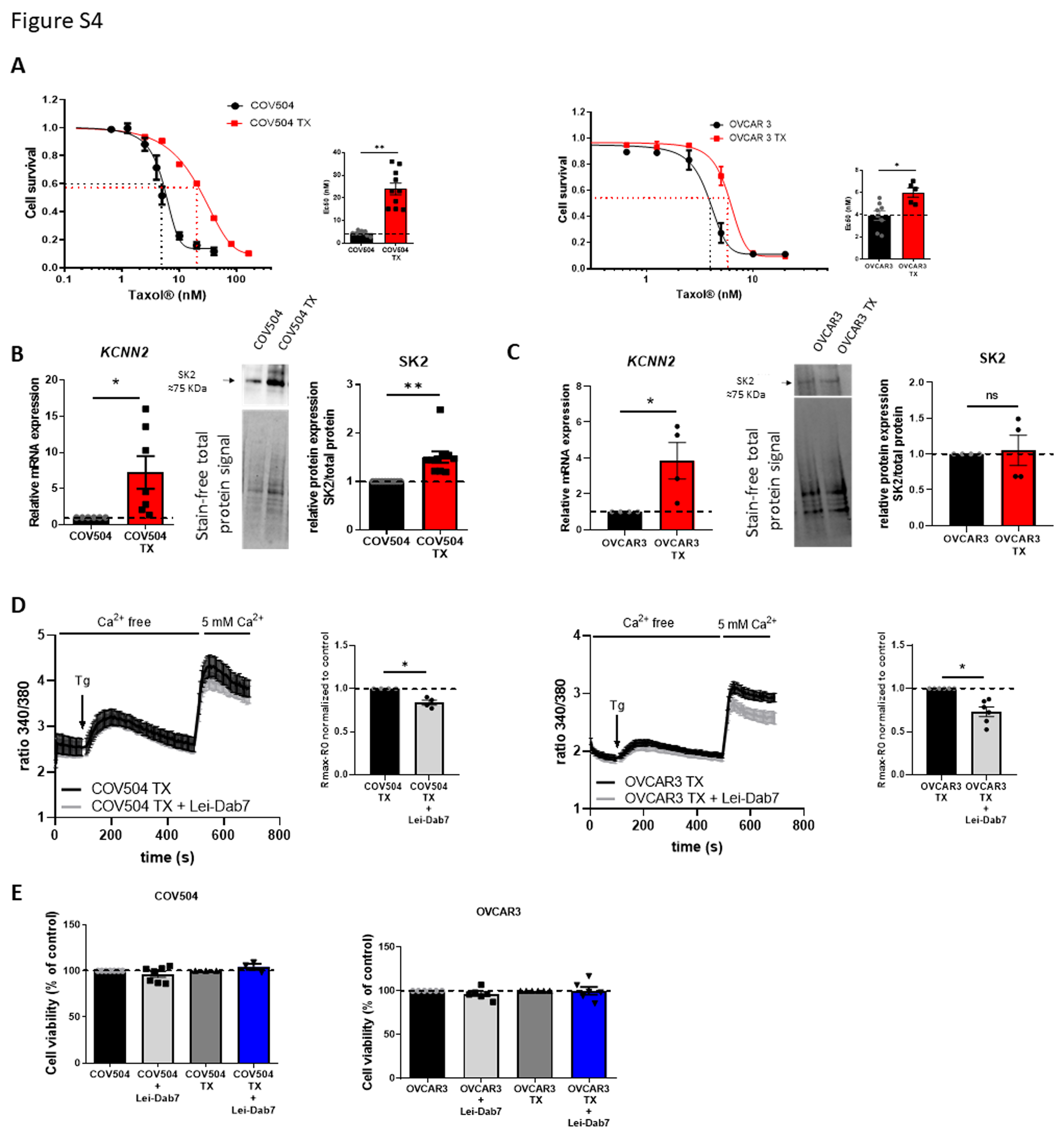

Supplement: Supplementary file 1 — Fig. S1. Functional plasma membrane SK2 does not participate in the CCE, and siSK2 specifically decreases KCNN2 but not KCNN1 or KCNN3. Fig. S2. In OVCAR3, LPA treatment increases KCNN2 mRNA levels without major impact on protein level and promotes the loss of sensitivity of SOCE to Lei‐Dab7. Fig. S3. Effect of LPA treatment on expression of KCNN1, KCNN2 and KCNN3 in COV504 and OVCAR3 cells and effect of SOCE inhibition on LPA‐treated cell migration. Fig. S4. Characterization of Taxol® chemoresistant sublines and effects of Lei‐Dab7 on SOCE and cell viability. Fig. S5. CyPPA, a SK2 and SK3 activator significantly decreases Taxol® resistance in COV504 and OVCAR3 cells. [file MOL2-18-1853-s001.zip › figure S4.TIF]

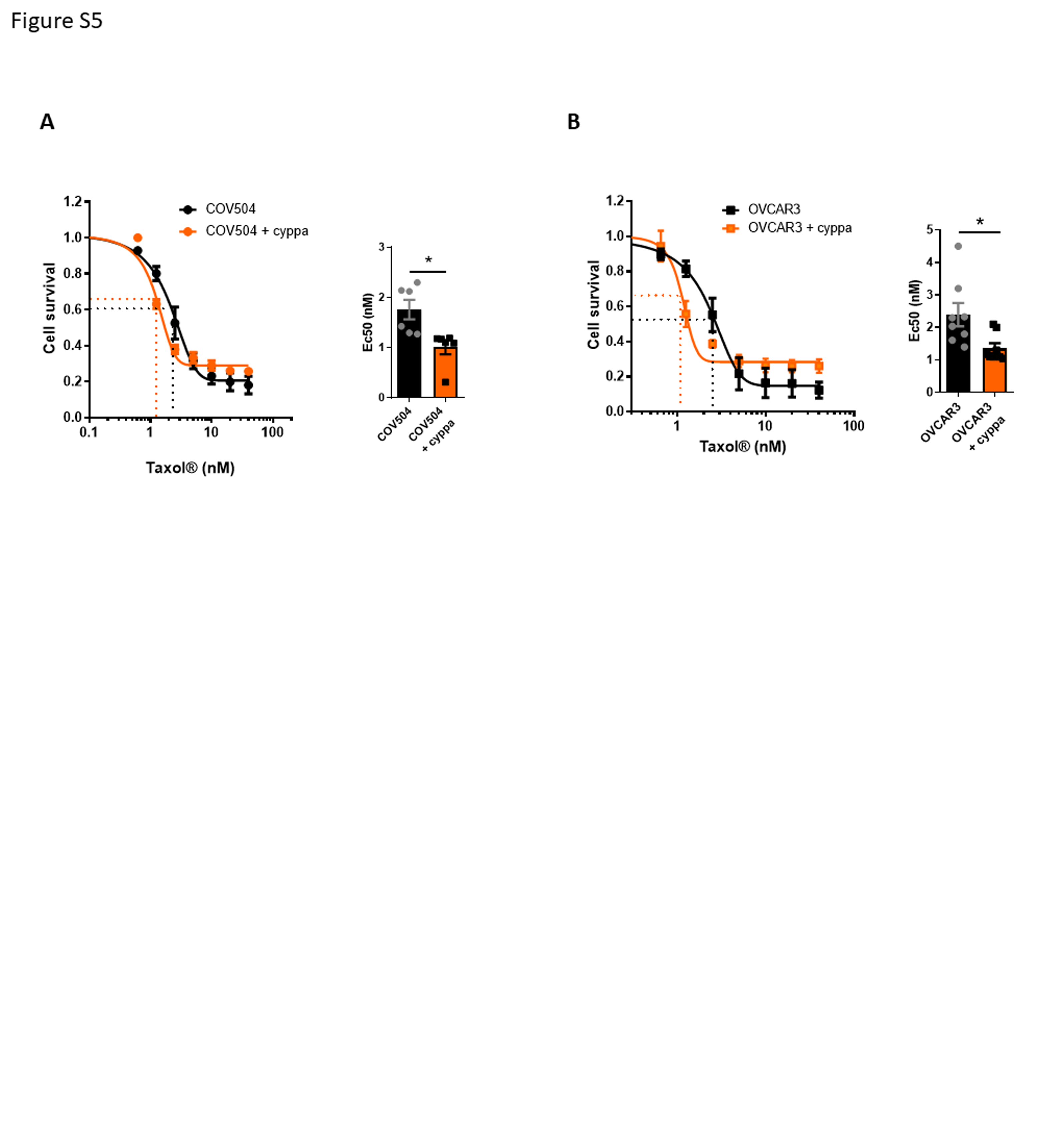

Supplement: Supplementary file 1 — Fig. S1. Functional plasma membrane SK2 does not participate in the CCE, and siSK2 specifically decreases KCNN2 but not KCNN1 or KCNN3. Fig. S2. In OVCAR3, LPA treatment increases KCNN2 mRNA levels without major impact on protein level and promotes the loss of sensitivity of SOCE to Lei‐Dab7. Fig. S3. Effect of LPA treatment on expression of KCNN1, KCNN2 and KCNN3 in COV504 and OVCAR3 cells and effect of SOCE inhibition on LPA‐treated cell migration. Fig. S4. Characterization of Taxol® chemoresistant sublines and effects of Lei‐Dab7 on SOCE and cell viability. Fig. S5. CyPPA, a SK2 and SK3 activator significantly decreases Taxol® resistance in COV504 and OVCAR3 cells. [file MOL2-18-1853-s001.zip › figure S5.TIF]

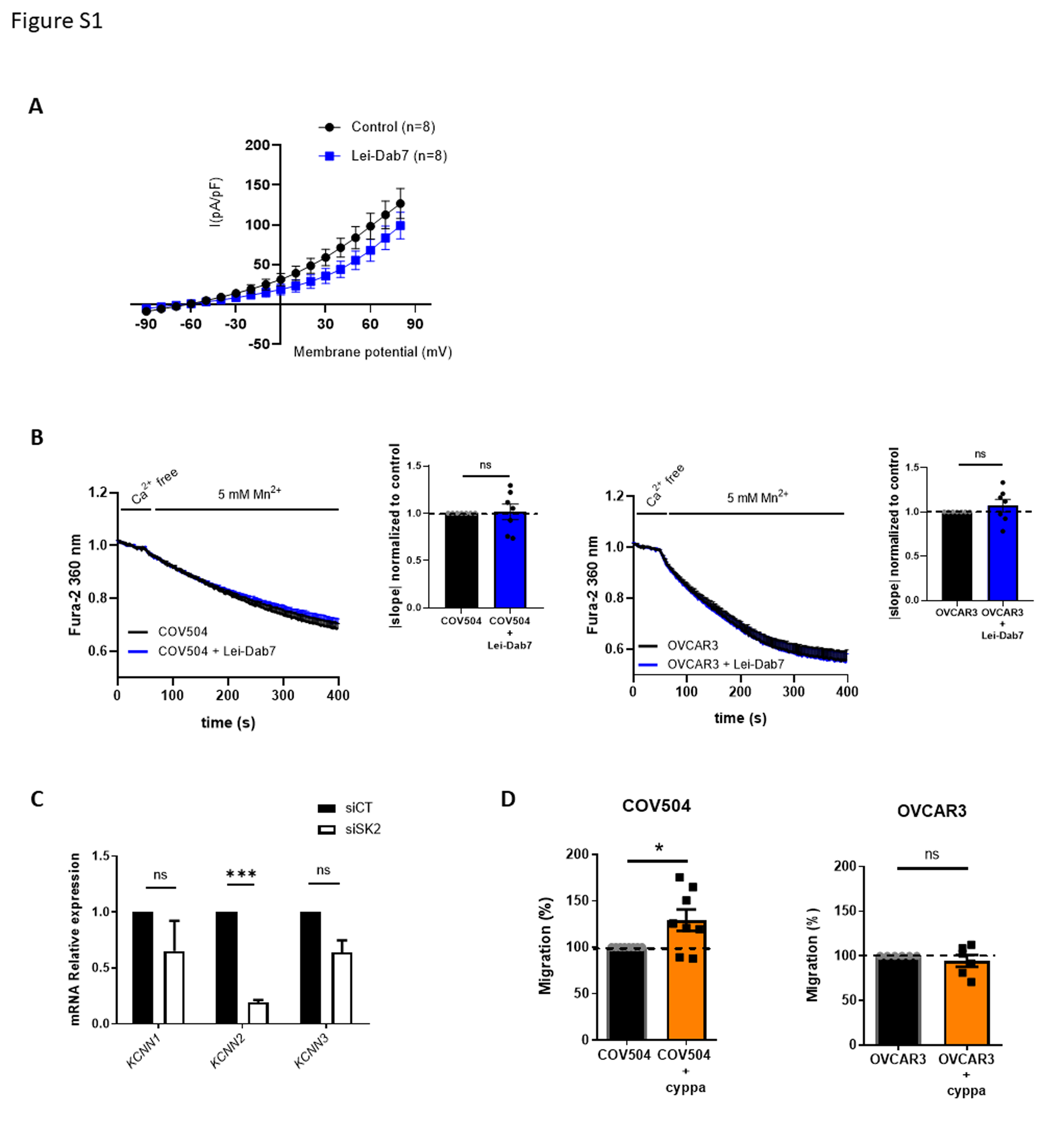

Supplement: Supplementary file 1 — Fig. S1. Functional plasma membrane SK2 does not participate in the CCE, and siSK2 specifically decreases KCNN2 but not KCNN1 or KCNN3. Fig. S2. In OVCAR3, LPA treatment increases KCNN2 mRNA levels without major impact on protein level and promotes the loss of sensitivity of SOCE to Lei‐Dab7. Fig. S3. Effect of LPA treatment on expression of KCNN1, KCNN2 and KCNN3 in COV504 and OVCAR3 cells and effect of SOCE inhibition on LPA‐treated cell migration. Fig. S4. Characterization of Taxol® chemoresistant sublines and effects of Lei‐Dab7 on SOCE and cell viability. Fig. S5. CyPPA, a SK2 and SK3 activator significantly decreases Taxol® resistance in COV504 and OVCAR3 cells. [file MOL2-18-1853-s001.zip › figureS1.TIF]

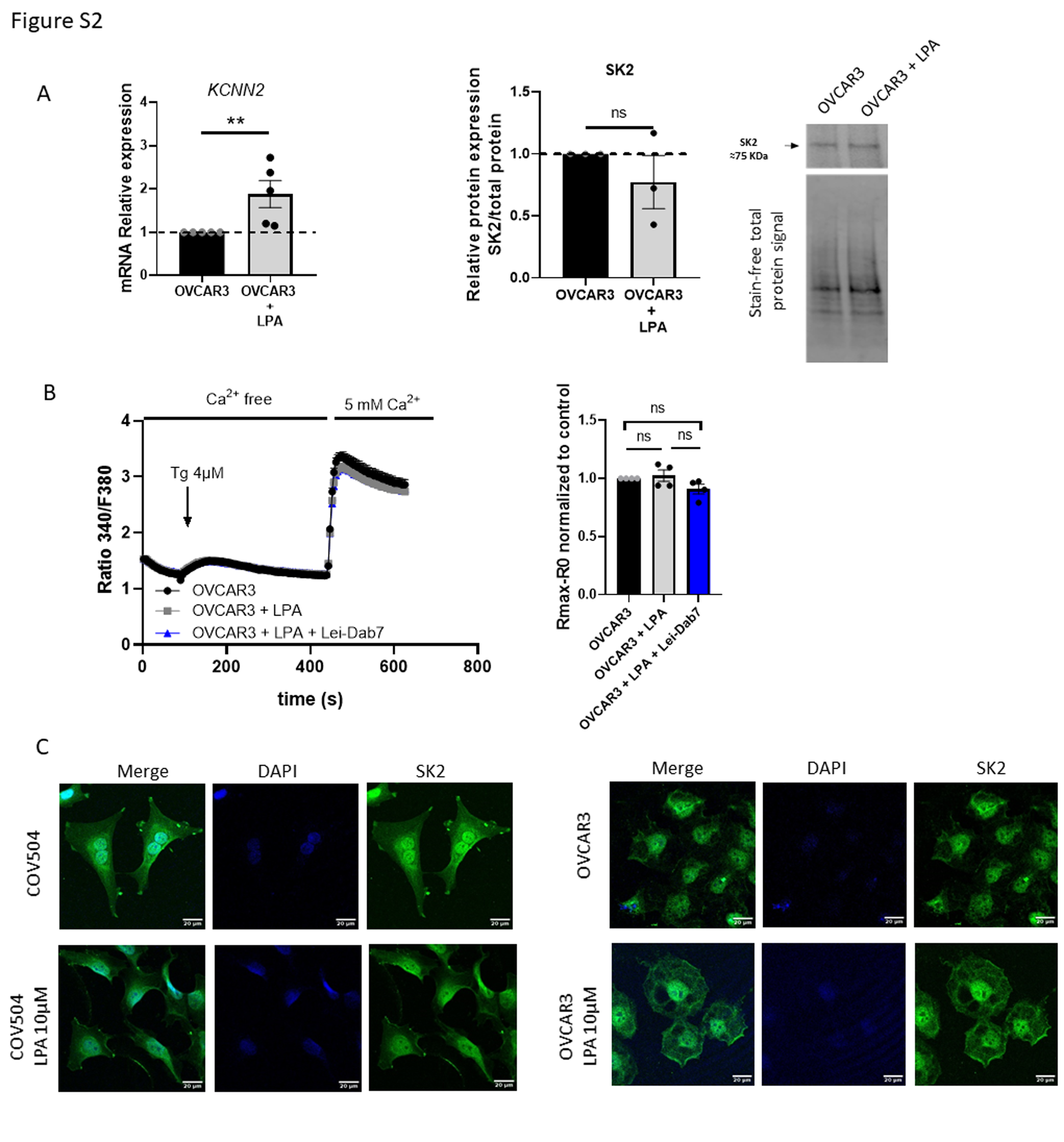

Supplement: Supplementary file 1 — Fig. S1. Functional plasma membrane SK2 does not participate in the CCE, and siSK2 specifically decreases KCNN2 but not KCNN1 or KCNN3. Fig. S2. In OVCAR3, LPA treatment increases KCNN2 mRNA levels without major impact on protein level and promotes the loss of sensitivity of SOCE to Lei‐Dab7. Fig. S3. Effect of LPA treatment on expression of KCNN1, KCNN2 and KCNN3 in COV504 and OVCAR3 cells and effect of SOCE inhibition on LPA‐treated cell migration. Fig. S4. Characterization of Taxol® chemoresistant sublines and effects of Lei‐Dab7 on SOCE and cell viability. Fig. S5. CyPPA, a SK2 and SK3 activator significantly decreases Taxol® resistance in COV504 and OVCAR3 cells. [file MOL2-18-1853-s001.zip › figureS2.TIF]
